# NUNDO: a numerical model of a human torso phantom and its application to effective dose equivalent calculations for astronauts at the ISS

**DOI:** 10.1007/s00411-014-0560-7

**Published:** 2014-08-15

**Authors:** Monika Puchalska, Pawel Bilski, Thomas Berger, Michael Hajek, Tomasz Horwacik, Christine Körner, Pawel Olko, Vyacheslav Shurshakov, Günther Reitz

**Affiliations:** 1 Institute of Nuclear Physics, Polish Academy of Sciences, 31-342 Kraków, Poland; 2 Applied Physics, Chalmers University of Technology, 41296 Gothenburg, Sweden; 3 Institute of Aerospace Medicine, German Aerospace Centre, 51147 Cologne, Germany; 4 Institute of Atomic and Subatomic Physics, Vienna University of Technology, 1020 Vienna, Austria; 5 Present Address: Department of Nuclear Safety and Security, International Atomic Energy Agency, 1400 Vienna, Austria; 6 Institute of Biomedical Problems, 123007 Moscow, Russian Federation

**Keywords:** Effective dose equivalent, International Space Station, Space radiation environment, Space dosimetry, Phantom experiments

## Abstract

The health effects of cosmic radiation on astronauts need to be precisely quantified and controlled. This task is important not only in perspective of the increasing human presence at the International Space Station (ISS), but also for the preparation of safe human missions beyond low earth orbit. From a radiation protection point of view, the baseline quantity for radiation risk assessment in space is the effective dose equivalent. The present work reports the first successful attempt of the experimental determination of the effective dose equivalent in space, both for extra-vehicular activity (EVA) and intra-vehicular activity (IVA). This was achieved using the anthropomorphic torso phantom RANDO^®^ equipped with more than 6,000 passive thermoluminescent detectors and plastic nuclear track detectors, which have been exposed to cosmic radiation inside the European Space Agency MATROSHKA facility both outside and inside the ISS. In order to calculate the effective dose equivalent, a numerical model of the RANDO^®^ phantom, based on computer tomography scans of the actual phantom, was developed. It was found that the effective dose equivalent rate during an EVA approaches 700 μSv/d, while during an IVA about 20 % lower values were observed. It is shown that the individual dose based on a personal dosimeter reading for an astronaut during IVA results in an overestimate of the effective dose equivalent of about 15 %, whereas under an EVA conditions the overestimate is more than 200 %. A personal dosemeter can therefore deliver quite good exposure records during IVA, but may overestimate the effective dose equivalent received during an EVA considerably.

## Introduction

Astronauts living and working on-board the International Space Station (ISS) at altitudes of about 400 km are exposed to radiation levels that are up to two orders of magnitude higher than at sea level. The main radiation hazards are due to galactic cosmic radiation (GCR) and due to protons and electrons of the radiation belt in the South Atlantic Anomaly (SAA). GCR consists of 98 % protons and heavy ions (baryon component), with energies from several tens up to 10^12^ MeV or more, and 2 % electrons and positrons (lepton component). The baryon component is composed of 87 % protons, 12 % helium ions (alpha particles) and 1 % heavy ions. Inside the ISS, a secondary radiation field with a significant contribution of neutrons is produced, due to nuclear interactions of the GCR with the atoms of the shielding material and the human body. The contribution of these sources to the radiation hazard to astronauts varies with the altitude, the solar activity and the local shielding by the ISS itself (NCRP 2000).

In the ISS orbit of 50° inclination, the geomagnetic field provides sufficient shielding to prevent high exposures due to solar energetic particle events and hence tissue reactions (deterministic effects). However, exposures by GCR may cause stochastic effects such as cancer or effects on the central nervous system. For determination of radiation risk on human health, it is necessary to derive quality factors and effective dose equivalent, which is the primary quantity in evaluating risk for health detriments from ionizing radiation in radiological protection in space. For this, one needs to know both, absorbed doses and linear energy transfer (LET) spectra in the organs of the body. Since organ doses cannot be measured directly in humans, the effective dose equivalent has to be determined by applying a suitable anthropomorphic phantom equipped with detector systems. Such a phantom was placed in the European Space Agency (ESA) MATROSHKA (MTR) facility (Reitz and Berger 2006; Dettmann et al. 2007), which was designed and built under the leadership of the German Aerospace Center (DLR). As phantom the RANDO^®^ (The Phantom Laboratory, Salem, NY, USA) was selected and equipped with a set of thermoluminescent detectors (TLDs) and plastic nuclear track detectors (PNTDs). The MATROSHKA facility was exposed for more than 1 year outside, and further on two times inside the ISS, thereby simulating an astronaut performing an extra-vehicular activity (EVA) and an intra-vehicular activity (IVA). Prior to these experiments, the effective dose equivalent was only measured once, with the human torso phantom Fred-1 placed inside the Space Shuttle during the STS-91 mission to the Russian Space Station MIR (Yasuda et al. 2000; Yasuda 2009), but was never measured outside a spacecraft.

In the present paper, the NUmerical model of the RANDO^®^ phantom (NUNDO) (Puchalska 2008) is reported as a suitable tool for organ dose and effective dose equivalent calculations. The procedure of the organ dose and the effective dose equivalent calculations is briefly introduced for the three MATROSHKA experiments for the years 2004 to 2009, outside and inside the ISS, based on measurements of PNTDs and TLDs. PNTDs were provided by the Oklahoma State University (OSU) and analysed/evaluated by DLR; TLDs were provided and analysed by the Institute of Nuclear Physics in Krakow, Poland, the Institute of Atomic and Subatomic Physics in Vienna, Austria, and DLR, Cologne, Germany. Whereas the paper Reitz et al. (2009) suffered from limited data sets (only doses and LET spectra for some organs are reported), the present paper uses the latest TLD results based on the publication Berger et al. (2013) and data from a complete set of LET spectra measured in PNTDs and presents doses for all organs and effective dose equivalents.

## Materials and methods

### The MATROSHKA facility

MATROSHKA is an ESA facility designed and built by DLR and flown from 2004 to 2011 on-board the ISS (Reitz and Berger 2006; Dettmann et al. 2007). MATROSHKA was designed to estimate the organ doses to astronauts inside the ISS and during an EVA, in order to improve the assessment of radiation risks in future space missions. For this purpose, an anthropomorphic human phantom (RANDO^®^), typically used in radiotherapy for dose verification, equipped with numerous radiation detectors (including TLDs (1,634 measurement points), NTDPs, silicon detectors, scintillators and a tissue equivalent proportional counter), and was exposed outside the Russian module Zvezda from 26 February 2004 up to 18 August 2005. This phase of the experiment was called MATROSHKA-1 (MTR-1). A carbon fibre container, with an average mass shielding of ~0.5 g/cm^2^, simulated the shielding distribution of an astronaut’s EVA suit. The second and the third phases of the experiment were performed inside two different segments of the ISS, the Pirs Docking Compartment (MTR-2A) and the Zvezda Service Module (MTR-2B), from 5 January 2006 to 7 December 2006 and from 18 October 2007 to 18 March 2009, respectively (Berger et al. 2013). In the present paper, only data from TLDs and NTDPs are reported.

### The RANDO^**®**^ phantom

**Fig. 1:**
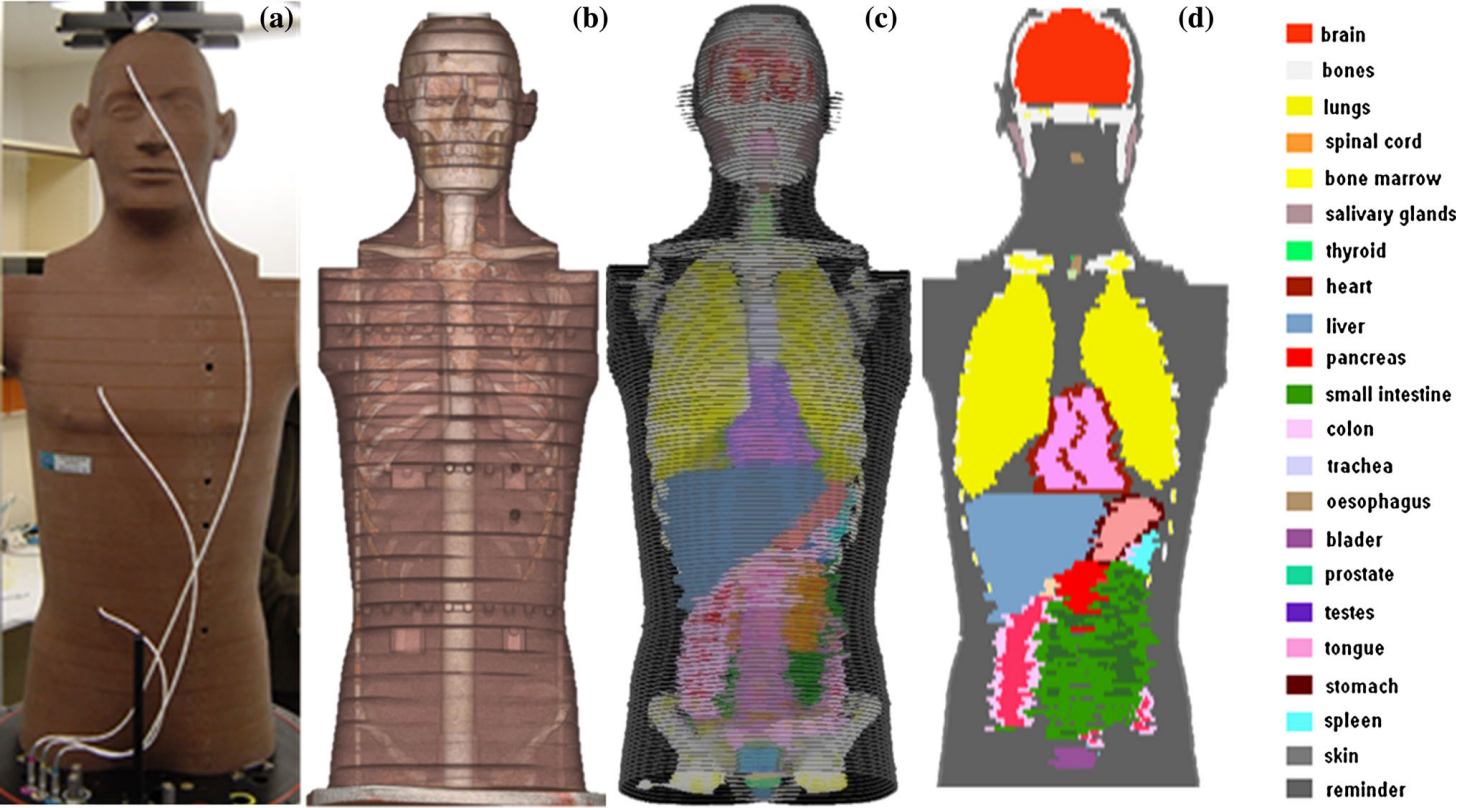
**a** Front view of RANDO phantom; **b** CT scan of the phantom; **c** and **d** the numerical voxel phantom NUNDO; organs and tissues are represented by different colours, and not all organs are visible

The RANDO^®^ phantom is an upper torso made of a natural human skeleton embedded in a tissue equivalent material (polyurethane) simulating soft and muscle tissues (*Z*_Eff_ = 7.4; *ρ* = 1.05 g/cm^3^). Polyurethane has an effective atomic number of 7.6 and a mass density of 0.997 g/cm^3^. A material with a lower effective atomic number of 7.1 and almost three times lower mass density, equal to 0.352 g/cm^3^, was used to simulate the lungs (*Z*_Eff_ = 7.4; *ρ* = 0.32 g/cm^3^). The phantom torso is 84 cm in height with a maximal width of 40 cm and a maximal depth of 22 cm. The phantom torso is shown in Fig. 1a, and the element composition is given in Table 1.

**Table 1:** Element composition (percentage by weight) of the RANDO^®^ phantom (The Phantom Laboratory, Salem, NY, USA)

Element	Soft tissue	Lungs
Carbon	67.78	70.74
Oxygen	20.31	21.28
Hydrogen	9.18	5.97
Nitrogen	2.50	1.9
Antimony	0.22	0.1

### Modelling of the numerical RANDO phantom (NUNDO)

The innovative part of this work was development of the numerical voxel model NUNDO of the RANDO^®^ phantom and its application to organ dose calculations. For this purpose, computer tomography (CT) scans of the RANDO^®^ phantom were performed (see Fig. 1b). A region-of-interest routine, implemented in the public domain Java™ image processing and analysis software *ImageJ* (Schneider et al. 2012), was used for image segmentation into clusters representing bones, lungs and soft tissues, and to fill each organ with a unique index value. Thus, the organ index image was achieved containing a 401 by 401 matrix, in which the Hounsfield numbers (Hounsfield 1973) were replaced by integers corresponding to the organ index value. For each CT slice, a file was created and named by the slice number. The final voxel model is then a three-dimensional array of 401 by 401 by 169 voxels, in which the resolution in the transversal plane is 1 mm per pixel and in the vertical plane is 5 mm per pixel. The total number of voxels is equal to 2.7 × 10^7^.

Since the physical RANDO phantom does not contain all the specific radiosensitive organs and tissues given by ICRP Report 103 (2007), the soft tissue material was replaced by the corresponding organs. This was performed by scaling the organs from the human phantom Zubal (Zubal et al. 1994) to the dimensions of the RANDO^®^ phantom. The NUNDO model is shown in Fig. 1c, d.

Table 2 compares the masses of the defined organs of the NUNDO voxel phantom, calculated by multiplying the volume of the voxel organs and their mass densities, with the ICRP Reference Man values (ICRP 2002). Using the skin density of approximately 1.1 g/cm^3^, the reference body surface area of 1.90 m^2^ and the total skin mass of 3,300 g for the reference male, the reference skin thickness (epidermis and dermis) can be estimated to be approximately 1.6 mm for the adult male (ICRP 2009). Hence, the skin of the NUNDO phantom is represented by 2 voxel layers, of 1 mm each, wrapping the phantoms’ exterior. Note that in Reitz et al. (2009), the skin thickness was 3 mm, following the medical sources.

**Table 2:** Mass of the NUNDO phantom organs compared to the ICRP reference man (ICRP 2002)

Organ	Mass (g)	
	NUNDO	ICRP 2002
Bladder	45	50
Brain	1,239	1,450
Breast	25	25
Colon	322	370 (73)
Oesophagus	34	40 (6)
Gonads	31	35 (12)
Heart	344	330 (55)
Kidneys	302	310 (80)
Liver	1,623	1,800 (390)
Lungs	1,720	1,200 (322)
Pancreas	128	140 (39)
Salivary glands	72	85
Small intestines	613	650 (78)
Spleen	157	150 (44)
Stomach	159	150 (46)
Thymus	24	25 (9)
Thyroid	19	20 (7)

In brackets, if available, the standard deviations for populations are shown (ICRP 2002)

The agreement for the inner organs, except lungs, with the Reference Man (ICRP 2002) is very good. A larger difference was observed for the lungs (~40 %), which is the result of the individual diversity for population. The RANDO^®^ phantom’s lung material closely mimics the density of lungs in a median respiratory state. The moulded lungs are handshaped and fitted to naturally fill the rib cage. Natural human skeletons are used, which are not always of the same size and shape. Also, many skeletons reflect natural human characteristics such as lack of symmetry and distorted joints (The Phantom Laboratory, Salem, NY, USA).

### Detector systems

The RANDO^®^ phantom is built up of 33 slices with a thickness of 2.5 cm each. In each slice horizontal cut-outs were milled to accommodate up to 5,800 TLDs fixed in polyethylene tubes. A total of 354 tubes allow a total number of 1,634 measurement positions inside the phantom, arranged in a way that the TLDs are positioned in a 2.54-cm orthogonal grid. The phantom was covered by a Nomex^®^ hood and poncho, which were also filled with TL detectors to calculate skin doses. TLDs are used to determine the dose for radiation with an LET below 10 keV/µm, where the TLD efficiency is equal to unity (Berger and Hajek 2008). The dose in the entire relevant high-LET range above 10 keV/µm can be measured by PNTDs with high efficiency. The combination of the passive TLDs and PNTDs is a commonly used technique in space dosimetry (Reitz 1994; Benton et al. 2002; Vanhavere et al. 2008; Straube et al. 2010). Due to limited space inside the phantom, detector packages containing PNTDs and TLDs were placed inside polyethylene boxes only at positions of selected organs and in the poncho. Data obtained with these detectors were used to calculate the relevant quality factors. The respective procedure is given in Reitz et al. (2009), where the PNTD data were provided by Johnson Space Center (NASA-JSC). The data from PNTDs presented here were determined from the detectors provided by OSU and evaluated and analysed at DLR.

Within the MATROSHKA facility, all participating laboratories providing data for the phantom depth dose distribution used TLDs based on lithium fluoride, activated with magnesium and titanium (^7^LiF:Mg,Ti). The measured signals were converted to units of absorbed dose in water through calibrations performed with secondary-standard gamma-ray radiation sources (^60^Co and ^137^Cs). The parameters for the detector preparations, readout and the quantification method of the TL signal are given in Reitz et al. (2009) and Berger et al. (2013).

The long-term stability of the TLD signal (fading) was studied by Bilski et al. (2013). The results revealed that for the properly oven-annealed LiF-TLDs, fading is not a significant problem. For measuring periods longer than a year, almost all measured doses were within 10 % deviation from the true values, while more than 80 % of the results show deviations smaller than 5 %.

### Three-dimensional dose distribution model

Three-dimensional (3D) continuous dose distribution models were created by interpolating between 1,634 discrete measured grid points (measured with TLDs in a 2.5-cm grid) (Berger et al. 2013). For interpolation, the inverse distance-weighted method, based on Shepard’s method (Shepard 1968) applying Liszka’s modifications (Liszka 1984), was used. This interpolation method is based on the assumption that the interpolated value should be influenced most by the nearby points and less by the more distant points. The dose at point *i*, *D*_*i*_, is the weighted average of grid point doses and is calculated by:

1 $$ D_{i} = \frac{{\sum\limits_{j = 1}^{N} {w_{j} \cdot D_{j} } }}{{\sum\limits_{j = 1}^{N} {w_{j} } }} $$

where *N* is the number of grid points, *D*_*j*_ is the dose value at grid point *j* and *w*_*j*_ is a weight factor assigned to each grid point expressed by:

2 $$ w_{j} = \frac{1}{{(R_{j}^{2} + \delta^{2} )^{{\frac{\beta }{2}}} }} $$

where *β* is a positive real number controlling the smoothness of interpolation (*β* = 2 by default), *R*_*j*_ is the distance between the grid point and the interpolated point and *δ* ^2^ is the measurement error (7 %). The weight assigned to each grid point diminishes as the distance from the interpolated point increases. The weight factor is normalized and thus that the weights sum up to unity.

Due to the high contribution of low-energetic electrons for the less shielded MTR-1 EVA exposure, a steep dose gradient from the skin layer towards the inner-body layers of around 80 % was observed (Reitz et al. 2009). Hence, for the interpolation from the measurement of the body layer to the first measuring point inside the phantom, an exponential attenuation function was adopted:

3 $$ D_{i} = D_{j} + D_{\text{skin}} \cdot \exp \left( {\frac{{x_{0} - R_{j} }}{t}} \right) $$

where *x*_0_ and *t* are parameters determining the slope of the dose decrease (*x*_0_ = −0.16; *t* = 1.26*)*, *D*_skin_ and *D*_*j*_ are the doses measured at the body surface and at the closest measurement point inside the phantom (~1 cm), respectively.

### Calculation of organ dose

Combining the 3D continuous dose distribution model and the NUNDO voxel model phantom, an average dose to the organ T was calculated according to Eq. 4.

4 $$ D_{\text{T}} = \frac{{\sum\limits_{i = 1}^{{N_{\text{T}} }} {D_{i} } }}{{N_{\text{T}} }} $$

where *D*_*i*_ is the dose value at point *i* that corresponds to a voxel *i* representing the organ T with the maximal number of voxels *N*_T_.

The TLD readings were corrected by subtracting the dose calculated from the LET spectra measured by the PNTDs for LET >10 keV/µm and weighted with the response function of the TLDs (Reitz et al. 2009). Each dose value *D*_*i*_ was calculated as the sum of the corrected experimental TLD absorbed dose and the dose calculated from the appropriate PNTD LET spectra. For other radiosensitive organs, where PNTD data were not available, an average correction factor of 1.07 was applied to the TLD measurements, based on the average ratio of the total organ dose measured with the combined TLD-PNTD method and the TLD organ dose (see Table 3).

**Table 3:** Average TLD organ dose rates for the whole LET spectrum and for LET <10 keV/μm (D_TDL_ and D_TDL-low_, respectively), average PNTD organ dose rates for LET >10 keV/μm (D_PNTD-high_) and total average organ dose rates (D_T_); total average organ dose equivalent rates for the whole LET spectrum and for LET >10 keV/μm ($\overline{H}_{\text{T}}$ and H_PNTD-high_, respectively) and mean quality factors ($Q_{T}$) for organs and locations where combination of TLDs and PNTDs were applied

	*D*_TLD_ (μGy/d)	*D*_TLD-low_ (μGy/d)	*D*_PNTD-high_ (μGy/d)	*D*_T_ (μGy/d)	*D*_T_/*D*_TLD_	*H*_PNTD-high_	$\overline{H}_{\text{T}}$ (μSv/d)	*Q*_T_ (μSv/d)
*MTR-1*								
Eye	527	507	37	544	1.03	479	986	1.8
Lung	266	252	27	279	1.05	407	659	2.4
Stomach	226	209	30	239	1.06	432	641	2.7
Kidney	199	186	23	209	1.05	313	499	2.4
Small Intestine	220	204	29	233	1.06	418	622	2.7
Skin	1,319	1,229	150	1,379	1.05	1,796	3,025	2.2
Poncho	587	497	150	647	1.10	1,796	2,293	3.5
*MTR-2A*								
Eye	209	193	28	221	1.06	381	574	2.6
Lung	181	165	28	193	1.07	391	556	2.9
Stomach	163	148	29	177	1.09	434	582	3.3
Kidney	154	142	22	164	1.07	329	471	2.9
Small Intestine	161	147	26	173	1.07	401	548	3.2
Skin	214	197	31	228	1.07	444	641	2.8
Poncho	223	205	31	236	1.06	444	649	2.8
*MTR-2B*								
Eye	173	156	30	186	1.08	396	552	3.0
Lung	172	155	30	185	1.08	430	585	3.2
Stomach	160	142	31	173	1.08	410	552	3.2
Kidney	168	150	30	180	1.07	393	543	3.0
Small Intestine	166	148	30	178	1.07	398	546	3.1
Skin	177	156	36	192	1.08	487	643	3.3
Poncho	173	152	36	188	1.09	487	639	3.4

PNTD data from detectors provided by OSU and evaluated/analysed by DLR

Relative precisions for dose rate values range between 4 and 8 % and for dose equivalent rate and *Q* values around 15 %

### Calculation of effective dose equivalent

The effective dose, *E*, is the sum of the equivalent doses, *H*_T_, in all the radiosensitive organs T, weighted by a dimensionless tissue weighting factor *w*_T_ that represents the relative contribution of the organ to the total detriment

5 $$ E = \sum\limits_{\text{T}} {w_{\text{T}} \cdot H_{\text{T}} } . $$

For radiation protection in space, the non-measurable equivalent doses are replaced by the organ dose equivalent $\overline{H}_{T}$ (ICRU 1993; NCRP 2000; ICRP 2013) which is calculated as a mean value over the whole organ of interest based on calculation of a dose equivalent at a point.

6 $$ \overline{H}_{\text{T}} = \int_{L} {Q\,(L)dD_{\text{T}} \,(L)} $$

where *dD*_T_ (*L*) is the contribution to the absorbed dose from a radiation component with an LET between *L* and *L* + *dL* and *Q* (*L*) is a dimensionless mean quality factor (ICRP 1991). Applying the organ dose equivalent, the respective quantity as defined in ICRP Report 123 (ICRP 2013) is henceforth called effective dose equivalent.

For these calculations, the *w*_T_ values were taken from the ICRP Recommendation 60 (ICRP 1991), which currently forms the basis of the EU legal radiation protection regulations. In 2007, a revised set of *w*_T_ values, selected by judgement on the basis of a broad range of experimental data, has been published in the ICRP Recommendation 103 (ICRP 2007). The changes in the tissue weighting factor *w*_T_ made by the Commission are as follows: the value for the breast increased from 0.05 to 0.12; that for gonads decreased from 0.20 to 0.08; those for bladder, oesophagus, liver and thyroid decreased from 0.05 to 0.04; a value of 0.12 is given in place of 0.05 for the remainder tissues; and an additional value of 0.01 is given for the brain and the salivary glands. In order to visualize the effect of these changes, in the present paper the effective dose equivalent was calculated using both, ICRP (1991) *w*_T_ values and ICRP (2007) *w*_T_ values.

The mean quality factors in selected organs, where PNTDs were applied, were calculated as the ratio between the total dose equivalent and the total organ dose (see Reitz et al. (2009) for details). For the calculation of the mean quality factors in all remaining organs, an interpolation was performed using the *Q* values received by LET spectra measurement in the selected organs, applying the mean shielding depth for the organs of interest in the NUNDO phantom (Matthiä et al. 2013).

## Results and discussion

The combined TLDs and PNTDs’ data for the MTR-1/2A/2B experiments for the organs and for the poncho detectors, which act as surrogate for a personal dosemeter of an astronaut, are presented in Table 3.

**Fig. 2:**
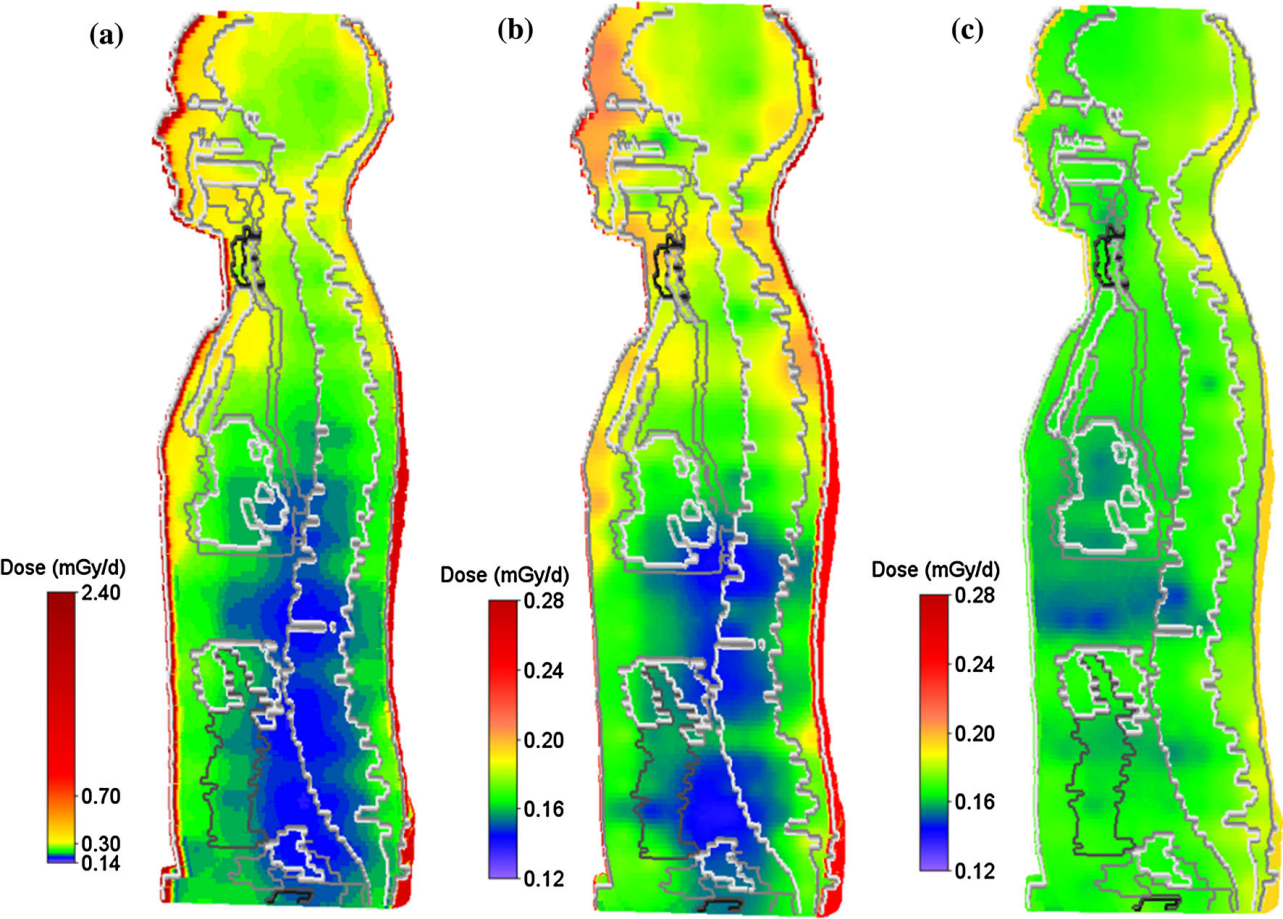
Continuous TLD dose distribution models combined with the numerical voxel phantom NUNDO for **a** MTR-1, **b** MTR-2A and **c** MTR-2B. Mean organ dose rates are calculated from these distributions (Tables 3, 4)

The average TLD organ dose rate calculated by folding the TLD 3D dose distribution model with the NUNDO voxel phantom (Fig. 2) is given by *D*_TLD_. *D*_TLD-low_ represents the average absorbed organ dose rate measured by TLDs for LET <10 keV/μm, and the *D*_*PNTD*-high_ represents the absorbed dose rate measured by PNTDs for LET >10 keV/μm. *D*_*T*_ is the total average organ dose rate given by the sum of *D*_TLD-low_ and *D*_PNTD-high_. The total daily organ dose equivalent rate ($\overline{H}_{\text{T}}$) and the mean quality factor (*Q*_T_) are based on the combination of TLD measurements and PNTD data. (see Reitz et al. 2009 for further details).

The mean quality factor ranges from 2.2 for the skin to 2.7 for stomach and small intestine for MTR-1. For MTR-2A, the mean quality factor for the skin is 2.8, while it is 3.3 for the stomach. For the MTR-2B exposure, the mean quality factors are quite homogenous and close to 3.1. The mean quality factor for stomach for MTR-1 agrees well with the value calculated by Zhou et al. (2010) based on NASA-JSC detectors.

The average organ dose rates, the average dose equivalent rates and the mean quality factors for all the radiosensitive organs, given by ICRP Report 103 (2007), for the MTR-1/2A/2B phantom torso experiments are summarized in Table 4.

**Table 4:** Average organ dose rates (D_T_), average organ dose equivalent rates ($\overline{H}_{\text{T}}$) and the mean quality factors ($Q_{T}$) calculated for all radiosensitive organs for MTR-1/2A/2B phantom torso facilities

Organ	*D*_T_ (µGy/d)			*Q*_T_			$\overline{H}_{\text{T}}$ (µSv/d)		
	MTR-1	MTR-2A	MTR-2B	MTR-1	MTR-2A	MTR-2B	MTR-1	MTR-2A	MTR-2B
Bladder	225 ± 13	173 ± 10	182 ± 10	2.4 ± 0.4	2.9 ± 0.4	3.1 ± 0.5	546 ± 82	507 ± 76	569 ± 85
Stomach	239 ± 14	177 ± 10	173 ± 10	2.7 ± 0.4	3.3 ± 0.5	3.2 ± 0.5	641 ± 96	582 ± 87	552 ± 83
Colon	245 ± 14	176 ± 10	181 ± 10	2.6 ± 0.4	3.2 ± 0.5	3.1 ± 0.5	636 ± 95	557 ± 84	565 ± 85
Red bone marrow	247 ± 14	180 ± 10	185 ± 11	2.5 ± 0.4	3.0 ± 0.5	3.1 ± 0.5	615 ± 92	543 ± 81	578 ± 87
Liver	250 ± 14	176 ± 10	178 ± 10	2.7 ± 0.4	3.3 ± 0.5	3.1 ± 0.5	669 ± 100	583 ± 87	557 ± 83
Reminder	260 ± 15	181 ± 10	179 ± 10	2.4 ± 0.4	2.9 ± 0.4	3.1 ± 0.5	618 ± 93	522 ± 78	559 ± 84
Oesophagus	275 ± 16	184 ± 11	178 ± 10	2.5 ± 0.4	3.0 ± 0.5	3.1 ± 0.5	691 ± 104	560 ± 84	556 ± 83
Lungs	279 ± 16	193 ± 11	185 ± 11	2.4 ± 0.4	2.9 ± 0.4	3.2 ± 0.5	659 ± 99	556 ± 83	585 ± 88
Bones	285 ± 16	190 ± 11	186 ± 11	2.4 ± 0.4	2.9 ± 0.4	3.1 ± 0.5	691 ± 104	558 ± 84	582 ± 87
Gonads	287 ± 16	183 ± 10	180 ± 10	2.2 ± 0.3	2.8 ± 0.4	3.1 ± 0.5	641 ± 96	512 ± 77	562 ± 84
Thyroid	316 ± 18	203 ± 12	180 ± 10	2.3 ± 0.3	2.9 ± 0.4	3.1 ± 0.5	736 ± 110	580 ± 87	564 ± 85
Brain	318 ± 18	199 ± 11	188 ± 11	2.3 ± 0.3	2.8 ± 0.4	3.1 ± 0.5	724 ± 109	561 ± 84	588 ± 88
Salivary glands	355 ± 20	203 ± 12	183 ± 11	2.3 ± 0.3	2.8 ± 0.4	3.1 ± 0.5	809 ± 121	571 ± 86	572 ± 86
Breast	485 ± 28	200 ± 11	179 ± 10	2.3 ± 0.3	2.8 ± 0.4	3.1 ± 0.5	1,107 ± 166	565 ± 85	559 ± 84
Skin	1,379 ± 79	228 ± 13	192 ± 11	2.2 ± 0.3	2.8 ± 0.4	3.3 ± 0.5	3,025 ± 453	641 ± 96	643 ± 97

For all organs, except lungs, skin and stomach, the D_T_ values were calculated by multiplying the TLD doses by an average correction factor of 1.07 (D_T_/D_TLD_ in Table 3). One-sigma uncertainties were calculated by error propagation

The results show a very steep, around 80 %, dose rate decrease from the skin (1,379 ± 79 µGy/d) towards the inner organs for MTR-1. This decrease is most pronounced in the lower part of the phantom (stomach, bladder) which is a consequence of higher body self-shielding and additional bottom shielding from the ISS.

The calculated dose rate for the skin for intra-vehicular exposure with MTR-2A (Pirs module) is 228 ± 13 μGy/d. In this case, the dose rate decrease from the skin towards the deeper located organs is <25 %. In contrast, for MTR-2B it is hard to see a decrease in dose rate towards the inner organs. The average skin dose rate for MTR-2B was calculated as 192 ± 11 µGy/d, which is approximately 15 % less than the average skin dose for MTR-2A. The difference in dose rate in the two different modules of the ISS results from a complex interplay of solar activity and shielding thickness: MTR-2B was exposed at lower solar activity than MTR-2A, which means that there was a higher contribution to the dose from the protons of the radiation belts and from GCR; on the other hand, the thicker shielding in case of MTR-2B compared with MTR-2A reduced the dose contributed by protons from the radiation belt. Deeper within the phantom, the solar modulation dominates any differences in dose, whereas closer to the surface of the phantom the shielding of the ISS module dominates, resulting in higher doses for MTR-2A (Berger et al. 2013).

Effective dose equivalent rates calculated either with *w*_T_ values from ICRP Recommendation 60 (ICRP 1991) or with those from ICRP Recommendation 103 (ICRP 2007) are shown in Table 5. The effective dose equivalent rate during an EVA (MTR-1) is 690 ± 33 μSv/d applying the *w*_T_ values from ICRP Report 60 and 722 ± 35 μSv/d when using *w*_T_ values from ICRP Report 103. In contrast, effective dose equivalent rates calculated for an IVA at different ISS modules based on ICRP Report 60 are 549 ± 27 μSv/d for MTR-2A and 566 ± 29 μSv/d for MTR-2B; while based on ICRP Report 103, they are 552 ± 26 μSv/d for MTR-2A and 566 ± 27 μSv/d for MTR-2B. For comparison, the effective dose equivalent rate onboard the vehicle during the short-term STS-91 mission was calculated as 418 µSv/d using *w*_T_ values based on ICRP Report 60 and 408 µSv/d using the ICRP Report 103 *w*_T_ values (Yasuda 2009). The small differences, 5 % for MTR-1 and <1 % for MTR-2A/B, in effective dose equivalent rate calculated based on ICRP Reports 60 and 103, demonstrate that the update of *w*_T_ values by ICPR 103 does not affect much the radiation risk estimates for stochastic effects in astronauts.

**Table 5:** Effective dose equivalent rates for *w*_T_ values taken from ICRP Report 60 (ICRP 1991) and from ICRP Report 103 (ICRP 2007)

	*E* (µSv/d)	
	(ICRP 1991)	(ICRP 2007)
MTR-1	690 ± 33	722 ± 35
MTR-2A	549 ± 27	552 ± 26
MTR-2B	566 ± 29	566 ± 27

One-sigma uncertainties were calculated by error propagation

Worth emphasizing is that the 15 % increase in the skin dose for MTR-2A compared to MTR-2B does not contribute much to the total effective dose equivalent for MTR-2A, as the contribution of the skin to the effective dose equivalent is only 1 %.

The use of the poncho detector set ($\overline{H}_{\text{T}}$ values from Table 3), a surrogate of a personal dosimeter worn by an astronaut, would overestimate the effective dose equivalent (Table 5) for an IVA by about a factor of 1.18 for MTR-2A and 1.13 for MTR-2B. During an EVA (MTR-1), the overestimation becomes larger than a factor of 3. When comparing the calculated here effective dose equivalent to the dose equivalent in the poncho detectors measured by NASA-JSC detectors (Zhou et al. 2010), this factor is about 1.9. The difference can be explained by the fact that the OSU/DLR detectors reported here were located at the outer surface of the detector package, thus representing a surrogate of a personal dosimeter, whereas the NASA-JSC detectors experienced an additional shielding of about 9.5 mm of plastic as they were located at the lower surface of the respective package.

## Conclusions

For the first time, a human torso phantom equipped with radiation detectors was used as part of the MATROSHKA facility to measure the doses at several locations inside and on the ‘skin’ of the phantom, which has been mounted outside the ISS simulating an astronaut during EVA. Following the outside exposure two measurement campaigns were performed inside the Russian modules of the ISS (Zvezda and Pirs). The measured depth dose profiles were combined with a numerical voxel model of the RANDO phantom (called NUNDO), in order to calculate organ and effective dose equivalents.

The main result of the present work is the depth dose profiles inside the phantom in the different exposure locations, which may be used to benchmark space radiation models and radiation transport calculations required for mission planning. Outside the station the depth dose gradient from the skin to the inner organs is very steep, demonstrating that measurements of a personal dosimeter dramatically overestimate the exposure of an astronaut, in the worst case by a factor of more than three. Exposures inside do not show this dramatic effect, but as lower the shielding thickness in inside exposures as steeper the gradient becomes. In the Pirs module (MTR-2A), the overestimation is about a factor of 1.18, whereas in the heavier shielded Zvezda module (MTR-2B) the overestimate is only a factor of 1.13.

Furthermore, it was shown that already in an outside exposure the self-shielding of the human body is very effective. The exposure of the various inner organs is comparatively homogeneous, and the effective dose equivalent is only less than 30 % higher than in an inside exposure.

So far, most of the measurements were performed with passive detector systems that do not provide time-resolved information. Therefore, future efforts aim at continuing with time-resolved measurements, to record the temporal pattern of the organ doses. In addition the results obtained so far are representing exposures at times of rather low solar activity. Accordingly, future measurements are planned at times of high solar activities, and the potential change of the depth dose distribution due to solar particle events should also be investigated. Note that solar particle events were absent during the measurement campaigns of the MTR facility.
